# Special Issue on Health, Physical Activity, and Performance in Youth

**DOI:** 10.3390/ijerph18178975

**Published:** 2021-08-26

**Authors:** Panagiota Klentrou

**Affiliations:** Department of Kinesiology, Brock University, St. Catharines, ON L2S 3A1, Canada; nklentrou@brocku.ca

## 1. Introduction

This Special Issue on “Health, Physical Activity, and Performance in Youth” was designed to be inclusive and diverse. With inclusivity in mind, “Youth” was defined broadly to encompass children, adolescents, and university students. Diversity was promoted through the issue’s wide spectrum of consideration, from youth with chronic conditions to young athletes. In summary, the 11 articles featured in this issue can be grouped into three categories: (a) physical activity and skeletal development, (b) physical activity behaviors and perceptions, and (c) young athletes and exercise performance.

## 2. Physical Activity and Skeletal Development

The first topic includes three papers on bone development. Constable et al. [1] examined whether the relationship between moderate-to-vigorous physical activity and total body less head bone mineral content (TBLH-BMC) is mediated by the free leptin index in prepubertal children. In this cross-sectional study, the authors used a four-way decomposition mediation analysis method, which showed that moderate-to-vigorous physical activity had a positive controlled direct effect on TBLH-BMC, independent of free leptin and possibly through mechanical loading, in both girls and boys [1]. However, since their participants were predominantly of normal weight, these observations may not apply to overweight and obese children at different stages of puberty [1]. In the same spirit, the longitudinal study by Ludwa et al. [2] followed a diverse sample of 180 children, aged 8–16 years, for 3 years, and showed that physical activity and BMI had a significant direct impact on bone properties and an additional indirect effect on muscle strength, which was the strongest predictor of bone speed with sound measurements. This study shows that the primary predictor is muscle strength, through the mechanical loading that drives bone development, whereas the impact of somatic maturity, physical activity, and BMI on bone development is also modulated by their effect on muscle strength [2]. Taking these observations one step further, Agostinete et al. [3] examined the mediating effect of lean soft tissue in association with somatic maturity and areal bone mineral density (aBMD) in a large sample size of 558 adolescents, who were grouped by sex and sport participation. They found that the association between somatic maturation and aBMD was mediated by lean soft tissue in both sexes, regardless of involvement in organized sports, also highlighting the important effect of muscle mass on bone [3]. Together, these studies confirm the role that physical activities that improve muscle mass and strength have on the development of bone mass during periods of growth.

## 3. Physical Activity Behaviors and Perceptions

The second topic is the most inclusive and diverse of the three topics, with five papers studying physical activity and health behaviors in diverse youth populations from elementary school children [4] and children with complex congenital heart disease [5], to middle- and high-school adolescent students [6] and adolescents living with human immunodeficiency virus (HIV) [7], to university students [8]. The collective conclusion of these articles is that positive encouragement and targeted physical activity interventions are important and vital to youth of all abilities and ages.

The pilot study by Farbo et al. [4] used accelerometers to investigate the effectiveness of a multiple recess school intervention on physical activity patterns in 157 younger (grades 1 and 2) elementary school children who participated in the LiiNK Project^®®^ (Let’s inspire innovation ‘N Kids). Students were assigned to either an intervention school, participating in 4 × 15 min unstructured (i.e., 60 min) outdoor recesses and one 15 min character development lesson daily, or a control school, participating in 2 × 15 min unstructured (i.e., 30 min) outdoor recesses daily, without the character development program [4]. The intervention students recorded more steps and time spent in moderate and vigorous physical activity (~900 more steps per day) than the control school students, suggesting that young children given 60 min of recess daily continue to increase physical activity patterns over those with 30 min of recess daily [4]. Longmuir et al. [5] used a qualitative approach to examine the physical activity perceptions of children with complex congenital heart disease and their parents to identify social and physical environment intervention targets. Through a series of semi-structured discussions, they concluded that, although both children and parents recognized the importance of physical activity, their activity-related uncertainty contributed to their inactive lifestyles, despite minimal restrictions from health professionals [5].

According to the systematic review by da Silva Cunha de Medeiros et al. [7], adolescents living with HIV have lower cardiorespiratory fitness, muscle strength, and body composition when compared to their uninfected peers, probably due to physical activity avoidance and other HIV-related adverse effects. However, due to the limited number of available studies in this population, there is an increased need for randomized clinical trials and observational studies to confirm the main results of this review [7]. The Olansky et al. [8] study investigated changes in body composition, energy expenditure, and dietary/energy intake from the end of the first to the end of the fourth year, in a sample of university students who were previously assessed during their first year. They found a positive increase in physical activity energy expenditure, between the end of the first year and the end of the fourth year, with no further changes in body mass, fat mass, and percentage body fat, which were increased during the first year in university [8]. Thus, despite the increase in physical activity in the last 3 years, students were unable to reverse the negative gains in body mass and adiposity observed in the first year, which may have negative health implications [8]. This result demonstrates that the development of targeted programs promoting a healthy transition to university life should be a priority for post-secondary institutions. 

On the other hand, performing physical activities and sports is not free of health risks and should be done with care. The study by Kim et al. [6] presents data from a cross-sectional web-based survey of health risk behaviors among a large sample of 60,040 Korean middle- and high-school students aged 12–17 years. They used a complex sample multivariable logistic regression model to identify factors related to tooth fracture experience in the past 12 months [6]. Interestingly, intensive physical exercise was one of the risk factors of tooth fractures; other risk factors included mental health conditions, as well as alcohol and tobacco consumption. The elevated risk of tooth fracture with intensive physical exercise was attributed to the higher likelihood of face-to-face impacts with a person or object observed in contact sport activities [6]. Therefore, a mouth guard is recommended to prevent tooth injuries in contact sports, which leads us to the third topic of this Special Issue.

## 4. Young Athletes and Exercise Performance

In the third topic, with a focus on athletes and exercise performance that includes papers on sport-related injuries [9], sports nutrition [10], and exercise testing [11], Snodgrass et al. [9] investigated whether static posture is associated with lower limb injury risk in young athletes. To this end, they analyzed the photographic posture data of 80 previous and 24 in-season lower limb injuries from 263 young male football players, aged 15 years or older, and from a range of competition levels in Australia. They found no association between previous injury and any postural variable [9]. In-season injury was significantly associated with previous injury and having a normal thoracic curve compared to kyphosis, but no other postural variables were identified. The authors concluded that static postural deviations observed in pre-season are not associated with non-contact lower limb injury risk, so there is no value in adding such observations in pre-season screening programs [9]. 

The paper by McKinlay et al. [10] examined whether two doses of whey protein consumed following high-intensity interval swimming had beneficial effects on muscle damage, inflammatory cytokines, and performance, compared both to an isocaloric carbohydrate beverage and a non-caloric water placebo in adolescent swimmers. Blood and 200 m performance measurements were repeated at 5 h, 8 h, and 24 h from baseline. Muscle soreness was assessed at 24 h. There were no differences in performance, creatine kinase activity, interleukin-6, and tumor necrosis factor-alpha between groups [10]. Self-reported muscle soreness was lower in the protein group compared to the water group, but not compared to the carbohydrate group. Importantly, interleukin-10, which is an anti-inflammatory cytokine, increased at 8 h from baseline in the protein group, while it decreased in both the carbohydrate and water groups. The authors concluded that post-exercise consumption of whey protein had no additional benefit on recovery indices following high-intensity swimming, compared to isocaloric amounts of carbohydrate beverage in adolescent swimmers, but that it may assist with the acute-inflammatory response to intense exercise in adolescent athletes [10]. 

Finally, Garcia-de-Frutos et al. [11] compared the maximal cycling power measured using three different work-to-rest protocols of high-intensity interval training (HIIT) with self-selectable load up to exhaustion, in 22 physically active, HIIT experienced, 19–24 years old males. The study used a cross-over design where participants completed one of three counterbalanced HIIT sessions per week. The intensity of the protocols was controlled using heart rate and blood lactate concentration levels. They confirmed that the work-to-rest ratio of the HIIT protocol can affect the test result, with the shorter work/rest time intervals allowing one to maintain a higher power for a longer time throughout the work session, without changes to blood lactate concentrations or a loss of power [11].

## 5. Conclusions

Collectively, the 11 studies in this Special Issue highlight the role of physical activity in improving fitness and the overall health of youth of all ages and abilities, that activity programs must focus on increasing energy expenditure and improving muscle mass and strength, consequently increasing bone mass and strength during periods of growth, and that injury prevention and adequate nutrition are highly recommended for young athletes who engage in competitive sports.

## Data Availability

Not applicable.
